# The destruction of the ‘Windrush’ disembarkation cards: a lost opportunity and the (re)emergence of Data Protection regulation as a threat to longitudinal research

**DOI:** 10.12688/wellcomeopenres.14796.1

**Published:** 2018-09-11

**Authors:** Andy Boyd, Matthew Woollard, John Macleod, Alison Park

**Affiliations:** 1 ALSPAC, University of Bristol, Bristol, BS8 2BN, UK; 2 CLOSER, UCL Institute of Education, London, WC1H 0NU, UK; 3 UK Data Archive, University of Essex, Colchester, CO4 3SQ, UK; 4 Population Health Science, University of Bristol, Bristol, BS8 2BN, UK

**Keywords:** GDPR, data protection, research archive, data retention, data repurpose, retrospective cohort study, follow-up, Windrush.

## Abstract

Historical records and the research databases of completed studies have the potential either to establish new research studies or to inform follow-up studies assessing long-term health and social outcomes. Yet, such records are at risk of destruction resulting from misconceptions about data protection legislation and research ethics. The recent destruction of the Windrush disembarkation cards, which potentially could have formed the basis of a retrospective cohort study, illustrates this risk. As organisations across Europe transition to the EU General Data Protection Regulation (GDPR), this risk is being amplified due to uncertainty as to how to comply with complex new rules, and the requirement under GDPR that data owners catalogue their data and set data retention and destruction rules. The combination of these factors suggests there is a new meaningful risk that scientifically important historical records will be destroyed, despite the fact that GDPR provides a clear legal basis to hold historical records and to repurpose them for research for the public good. This letter describes this risk; details the legal basis enabling the retention and repurposing of these data; makes recommendations as to how to alleviate this risk; and finally encourages the research and research-active clinical community to contact their ‘Data Protection Officers’ to promote safe-keeping of historical records.

## Disclaimer

The views expressed in this article are those of the author(s). Publication in Wellcome Open Research does not imply endorsement by Wellcome.

## Introduction

The UK state mistreatment of the ‘Windrush generation’ who migrated to the UK from the West Indies during the 1950s and 60s is a public scandal, but also illustrates a meaningful (re)emerging risk to longitudinal research. The scandal emerged from the ‘hostile environment’ ^1^ resulting from government policy to reduce UK immigration rates. Individuals legitimately living in the UK were forced to demonstrate their residency; while in parallel, officials destroyed—reportedly due to ‘Data Protection’ requirements ^2^ —the disembarkation records that could help prove citizenship status. Aside from their (disputed) utility to demonstrate citizenship, these disembarkation cards, a record of ~0.5 million migrants from a defined geographical location arriving in Britain following the end of the Second World War ^3^, could have been the starting point for a retrospective cohort study. With increasing realisation of record linkage strategies to enable retrospective and prospective follow-up, could a ‘Windrush Cohort’ have provided unique insights into the health and social outcomes of these migrants as they entered old age? Could such a cohort illustrate patterns of migration, community formation, economic and social integration and health outcomes within a population with distinct genomic and phenotypic characteristics and whom faced considerable socio-economic adversity? How would members of this community have felt about such a cohort? We may never know; while some equivalent records exist on a sub-set of this population, by destroying the disembarkation cards, this tantalizing possibility may be beyond reach. In this article we reflect on the value of repurposing historical records or historical research databases in long-term outcome studies; and then, assess the legal basis for retaining such records and repurposing them in this manner under the new EU General Data Protection Regulation (GDPR). We end by setting out provisional recommendations for clearer guidance and working practices that retain the potential for these records to inform future research, while retaining public acceptability.

## The value of repurposing historical records

Repurposing historical records to define retrospective cohorts or to allow long-term follow-up of clinical trials is not a trivial exercise, but has immense epidemiological value. Such studies effectively shortcut the passing of time: allowing the assessment of early exposures, including randomly allocated interventions, on outcomes decades later without waiting for those years to pass. Cohort examples include the Lothian Birth Cohorts ^4^, the Hertfordshire and Helsinki Cohorts ^5^, and the Boyd Orr Cohort ^6^, which have all repurposed historical records as a basis for contemporary studies. The Barry-Caerphilly ^7^, Sorrento ^8^, and Aberdeen ^9^ trial follow-up studies illustrate the potential to measure health outcomes long after the original interventions. The Historical Sample of the Netherlands ^10^ illustrates a variation on this theme: where registry records have been collated into a multi-generational longitudinal ‘spine’ linkable to other databases, an approach recognised as having potential to facilitate longitudinal research ^11^. These studies have made innovative use of historical records, established platforms for interdisciplinary research, and in turn, produced a wealth of research outputs (see Table 1). None of these would have been possible without the (sometimes accidental) preservation of the underlying, identifiable, historical records.

**Table 1.** Example longitudinal resources sampled from historical records.

Study	Location (City/Region, Country)	Historical records used for sampling	Sampling frame era
Lothian Birth Cohorts	Scotland, UK	School administered intelligence tests	1921 and 1936
*Aim:*		To assess cognitive change over the life-course	
*Notable* *findings/outputs:*		Identified that childhood cognitive ability accounts for half the variance in ability in older age	
Hertfordshire Cohort Study	Hertfordshire, UK	Midwifery registers	1931–1939
Helsinki Cohort Study	Helsinki, Finland	Child welfare clinic records	1934–1944
*Aim:*		To generate evidence to support the ‘fetal origins hypothesis’	
*Notable* *findings/outputs:*		Established association between early developmental conditions and adult health outcomes	
Boyd Orr Cohort	UK	Family Diet and Health’ survey	1937–1939
*Aim:*		To investigate early-life dietary exposures on adult health outcomes	
*Notable* *findings/outputs:*		1) Identified links between child diet and cancer outcomes 2) Demonstrated the impact of nutrition on subsequent inter-generational health, and of breastfeeding on later cardiovascular mortality	
Barry Caerphilly Growth Study	Wales, UK	Intervention trial records	1972–1974
Sorrento Maternity Hospital Study	Birmingham, UK	Intervention trial records	1979–1980
Aberdeen Folic Acid Supplementation Trial (AFAST)		Intervention trial records	1966–1967
*Aim:*		To conduct long-term follow-up of trials of nutritional manipulation during pregnancy to enable the examination of effects on outcomes in adult offspring	
*Notable* *findings/outputs:*		1) In Barry Caerphilly those given the intervention (free milk in pregnancy and early childhood) had lower Insulin-like growth factors (IGF-1) than the control group. 2) The Sorrento study found no evidence that nutritional supplements given to pregnant women are an important influence on adult disease risk; 3) In AFAST, findings suggest that suggest that maternal folic-acid supplement use is associated with changes in the DNA methylation of the offspring that persist for many years after exposure in utero.	
Historical Sample of the Netherlands	Netherlands	Birth, death, marriage certificates; population registers	1812–1920
*Aim:*		To produce a representative resource for demographic, social science and epidemiological research	
*Notable* *findings/outputs:*		Established a national ‘life history’ database which can be linked to other resources for diverse research applications	

## Re-emerging risks of a ‘bonfire of the records’

The risk of a ‘bonfire of the records’ ^12^ exists where ‘data protection’ concerns lead to the destruction of historical archives. The same risk also applies to existing cohort studies, particularly when: study participants are in transition (e.g. child participants reaching legal majority, or aging participants lacking the capacity for ongoing follow-up); studies face gaps in funding; intervention trials reach the end of their initial protocol; or when participant consent is no longer considered valid. A perceived ‘end’ of study could be coupled with pressure to destroy research databases or render the data anonymous. Even where anonymised records survive, these actions may preclude new data linkage opportunities, hamper integration into study consortia, or hinder assessments of long-term outcomes.

The risk of data destruction is re-emerging through the new EU GDPR and national implementations of the regulation ^13, 14^ Specifically, as we discuss below, through the potential for misinterpretation and as a result of the requirements within GDPR to audit information, compile catalogues of information with assigned data retention and destruction requirements. GDPR, like predecessor legislation, relates to ‘personal data’, i.e. data that can be related to specific living individuals. GDPR includes rephrased data retention requirements (GDPR Article 5(1)(e)), and new requirements that organisations systematically catalogue data assets (GDPR Article 30) and define retention and destruction rules for each type of asset (GDPR Recital 39). In practice, this means that universities, government departments and health bodies across Europe are conducting data audits, cataloguing data—including historical records and medical research databases—assessing their ‘lifespan’ and determining when they should be destroyed. Whether from sincere attempts to comply with new legislation, ignorance of historical data’s future research potential, or from pressure to clear shelf/server space, it is likely that historical records—equivalent to those used to establish the Boyd Orr, Lothian, Hertfordshire and Helsinki cohorts—are now under considerable threat of destruction. To counter this threat, it is important that the ethico-legal basis for retaining these records is clarified and communicated: here, we illustrate a case using UK Data Protection law as an example.

## The legal basis for retaining and repurposing historical records for research

The former UK Data Protection Act 1998 (DPA98) stated that “Personal data shall be obtained only for one or more specified and lawful purposes, and shall not be further processed in any manner incompatible with that purpose” (DPA98 Principle 2) and that data “shall not be kept for longer than is necessary for that purpose” (DPA98 Principle 5): suggesting that retaining and repurposing historical records is prohibited. However, the value of research is acknowledged in research exemptions (DPA98 s33); in regulatory guidance stating that “records selected for permanent preservation as archives, with a view to their use in historical or other research” is a legitimate ‘purpose’ within DPA98 ^15^, and a regulator endorsed code of practice stating: ^16^

“4.3.2 There is a danger that over-cautious interpretation of the Act may lead to the weeding, anonymising or destruction of files containing personal data that would otherwise be passed to the archives repository. An archivist’s ability within the Act permanently to retain personal and sensitive personal data for the purposes of research (see 4.2.1) should therefore be made clear to potential depositors. The legislation contains the necessary safeguards for depositors.“

If the Windrush archive was destroyed due to concerns around data protection (specifically the requirements of the DPA98, which was in force at that time), either these safeguards were not sufficiently communicated; or insufficient safeguards existed to protect against destruction from individuals who could not perceive the wider value of the records. The impetus for the new GDPR, along with the UK Data Protection Bill (DPA18), is to protect citizen interests in a digitized world where personal data are a ‘monetized’ commodity with both legitimate and transparent use and illegitimate and opaque use. Such protection is established in core principles (GDPR Article 5). Following extensive lobbying ^17^, GDPR recognises the benefits of research conducted for the public good and provides archive and research exemptions to the purpose and storage limitations (subject to Article 89(1) safeguards). This results in a permissive legislative framework for research and archiving, while raising data management standards and providing freedom of academic expression (Article 85(1)). GDPR recognises, in Article 5(1)(e), that any data can be used for research and stored as such for many years:

“personal data may be stored for longer periods insofar as the personal data will be processed solely for archiving purposes in the public interest, scientific or historical research purposes or statistical purposes in accordance with Article 89(1)”

The UK government have clarified that archiving is permitted within the proposed DPA18 ^18^. However, research data or archive records are not exempt from GDPR requirements to catalogue ‘information assets’ and set retention timescales. Therefore, the risk of records of research importance being destroyed comes from operational decisions made by administrative rather than research staff; and likely from a place of not fully understanding the research aspects of the new legislation.

## A call for clearer guidance

There is therefore an urgent need for operational guidance describing the options for retaining and repurposing this class of information for research purposes. The default should be that information with potential research/historical interest—a deliberately broad category—should be retained rather than destroyed. And that retention should be indefinite, albeit subject to periodic review where the benefits and risks of holding data can be assessed in a contemporaneous context. We argue this from the position that GDPR is not intended to curtail research, that research aiming to improve public goods typically enjoys strong public support, and the low risk to data subjects resulting from archiving or research follow-up. Following lobbying from the research community, the Information Commissioner’s Office (the UK Data Protection regulator) has issued guidance supporting this position ^19^. The research ethics community should produce new guidance for researchers designing new study (e.g. RCT) protocols, and new guidance for ethics committee members assessing them. Best practice standards (such as those evident in the findings of the ‘Understanding Patient Data’ taskforce in the UK) should be followed in order to develop clear and consistent public-facing language that explains the principles and reasons for retaining and repurposing records. There remains an open question as to how best communicate data repurposing, and how to engage the public in this activity. While the mechanism for this is likely to be context specific, we suggest that prior to repurposing comprehensive efforts should be made to engage data subjects, or where this is not possible, to test the public acceptability of the specific data reuse. We support the need that the retention of records is subject to meeting safeguarding requirements (GDPR Article 89) insofar as these do not limit future (and as yet unspecified) needs; and that repurposing records requires appropriate governance safeguards (e.g. research ethics approval with meaningful public input); and both should occur within socially acceptable frameworks ^20^.

We set out provisional recommendations for addressing this risk within Box 1, but consider that these need developing with professional (e.g. the Archives and Records Association and The National Archives in the UK) and public input. Resulting clear guidance should be communicated to institution ‘Data Protection Officers’—a staff role that all organisations must now have, as mandated under GDPR—to clarify the legal basis for retaining and repurposing records. Institutional and national archives could curate information from dormant studies (e.g. the UK Data Service’s ReShare online repository, or the University of Bristol’s data.bris archive). However, while such repositories could have value in reducing risks and increasing public acceptability, these will do little to address our primary concern that the research value of historical records is not clearly perceived.

## Conclusion

It is clear from the examples considered above that repurposed historical research and administrative records can make unparalleled contributions to driving improved scientific understanding and improving health and social policy. To neglect the safe keeping of these records will be to neglect this aspect of longitudinal research. We encourage researchers and research-active clinicians across Europe to contact their Data Protection Officer to alleviate this risk. The wider research community should work with regulators, archivists, data managers and other stakeholders to ensure long-term retention of these data in a manner that is fair and transparent to data subjects.

Box 1. **Recommendations for long-term retention and subsequent repurposing of records in a publicly acceptable manner**

1) That EU Data Protection regulators (referred to as national authorities in the GDPR) consider the risk of unnecessary record destruction and either:

i.   provide clear guidance to records managers regarding the permissive nature of GDPR for the long-term retention of personal data in archives, and potential for repurposing of these archives for research purposes;

Or

ii.   ratify a code of conduct providing this guidance that has been developed by appropriate national organizations.

2) That this precise scenario is communicated to key individuals; i.e. those likely to be conducting data audits and making decisions about retention schedules (e.g. all Data Protection Officers (a post mandated within GDPR), research managers, records managers, research ethics chairs and facilitators, research funders archivists).

3) That data retention decisions are recorded by those with responsibility for managing data (with oversight from Data Protection Officers), that decisions are internally audited and retained to ensure institutional memory of the value of specific records.

4) That where historical personal data are scheduled for destruction, and there is doubt regarding their retention value, that guidance is sought and due process is followed. It should be recognized that individuals may be confident there is ‘no doubt’ about the (lack of) value in a record: and that this can only be countered by raising the general appreciation of the value of these data. Consideration should be made at a national level as to the best means to achieve this in a transparent manner with input from diverse stakeholders. Options could include a panel of interdisciplinary experts to provide such guidance, or a system of public notifications where destruction is embargoed until a ‘consultation’ time period expires (i.e. a system akin to the UK land use planning permission system).

5) Both new and existing studies (both observational and interventional) shall (where practicable) inform potential participants that the personal data they provide will be retained for long periods (perhaps indefinitely with periodic retention reviews) and potentially repurposed within a given governance framework.

6) That research funders produce clear guidance on personal data retention and repurposing; and promote their funding mechanisms to support long-term archiving of important records.

7) That, prior to repurposing taking place, a code of practice is developed that establishes key principles. These principles should include guidance on:

i) the requisite safeguards;
ii) how representatives of the participant community are engaged and how their views are integrated into the research design in a meaningful manner;
ii) how participants are informed about repurposing (given that direct contact with all individuals may be impracticable) and if they are given the right to object;
iii) how the principle of fairness is retained during the repurposing of records for new research purposes

8) As a research and archive community we should learn from recent data scandals and consult the public on their understanding and expectations relating to retention and repurposing, and ensure appropriate safeguards are implemented to ensure continuing acceptability.

9) Information about archived records should be discoverable to the public (to ensure retention and repurposing is transparent) and researchers (in order to maximise appropriate use).
