# Supplementary material for: The destruction of the ‘Windrush’ disembarkation cards: a lost opportunity and the (re)emergence of Data Protection regulation as a threat to longitudinal research
Source: Wellcome Open Res. 2018 Sep 11;3:112. [Version 1] doi: 10.12688/wellcomeopenres.14796.1 (PMC6171557; doi:10.12688/wellcomeopenres.14796.1)
Supplement: Peer review history

# Peer review: Referee response for version 1

**DOI:** 10.21956/wellcomeopenres.16124.r33871

**Published:** 2018-10-02

**Authors:** Elizabeth Lomas

**Affiliations:** 1 iSchool, Department of Information Studies, University College London, London, UK

This open letter is submitted by a group of academics with specific expertise in the use of data for longitudinal studies. As such they offer a valuable perspective on record retention decisions and perceived risks influencing these decisions. In particular, they highlight the dangers posed by misinterpretations of the requirements of the new General Data Protection Regulation (GDPR) and Data Protection Act 2018 which together legislate for the management regimes for personal data in the UK. The authors make a call for further guidance on the correct application of data protection law to take account of archival considerations.  This review of the letter draws out some points for the authors further reflection. The review does not dispute the viewpoints and credibility of the piece but rather raises some issues for consideration. Overall it is the conclusion of the reviewer that it is important not only that this letter has been published but that the discussion contained within it is further publicly evolved beyond this piece. This review highlights some further guidance which is relevant to the discussion.

 The focus of this letter relates to the historical research and archival value of holding records through time. The letter uses the emotive case of the destruction of the ‘Windrush’ disembarkation records as a lens to evidence key issues. The authors rightly state that the use of these records might have enabled the identification of a particular cohort with a range of challenges worthy of special examination, for example in terms of their social, economic and health outcomes through time. Whilst the initial case for the value and retention of the landing cards of the so called ‘Windrush Generation’ is made persuasively within the piece, it would have been beneficial to discuss this in greater detail to develop the case that these records did merit permanent archival preservation. The National Archives does hold Board of Trade passenger lists (BT26) which provide some of the same data covered by the landing cards. These are given limited discussion on the website of The National Archives by Kershaw (2018) ^1^. Significantly, the passenger lists span a much narrower period from 1947-1953. However, the question arises as to whether from a historical/archival perspective the landing cards from this same period would have added significantly to the data captured by the passenger lists. I would have liked some further perspectives on the value of the cards for the period. Where multiple data sets/records exist, with overlapping information, researchers need to argue persuasively for the retention of each data set/record series. All data retention comes at a cost to the public purse and not everything can be kept so we do need more ongoing debates around our expectations for archiving. The authors were well placed to develop and evidence some of these arguments.

 In the Windrush case, the Home Office should have been best placed to comment on the current operational needs for retention.  Government Department officials will have known, or certainly should have known if tasked with this responsibility, that the landing cards could have been retained for operational purposes and as a separate consideration for permanent archival preservation. It appears that it was reasoned the cards were no longer required when considered in relation to the storage costs. A key issue in this example, from an operational perspective, is that it has been reported in the press that the records were still in current use when the disposal decision was made in 2009. There are balances to be struck in retention and destruction decisions. Any comment on the 2009 decision needs to be in the context of the operational requirements at the time of the evaluation. However, these records became of greater significance due to the Home Office later changing its rules in terms of the expectations of individuals to document their lives in the UK. The balance between State and individual recordkeeping responsibilities would merit a whole separate discussion. In regards to the archival selection processes it is important, and perhaps surprising to many, to note that the Home Office held the responsibility not only for the decisions concerning the current operational retention of these records but also the historical/archival selection decisions. This letter could have opened up this point to further consideration.  The National Archives does work closely with Departments to determine the records which need to be kept permanently. Operational Selection Policies set out the selection criteria (see http://www.nationalarchives.gov.uk/information-management/manage-information/selection-and-transfer/selecting-records/osp-number/ ) in conjunction with other overarching guidance which is in the public domain (e.g. TNA, 2016 ^2^). It is to be observed in terms of The National Archives’ collection policies, that traditionally there has been a greater emphasis on focusing resources on archiving policy documentation rather than necessarily on taking complete personal data sets. The letter strongly makes the case for considering in greater detail the value of personal data sets/record series as significant historical resources. The authors bring into the discussion further case examples of records with personal information, which have in some instances survived by accident rather than design. These records have subsequently been used for significant research purposes. The inclusion of a table of examples is particularly beneficially in providing an overview of these records. The focus on the value of different types of records provides a significant academic perspective which needs to be captured and considered more extensively and this could be done in relation to the published Operational Selection Policies which are the subject of public consultation. Therefore, I would suggest that the authors could make a stronger call for academic engagement with the Operational Selection Policies published by The National Archives and the related consultations processes. Various groups of stakeholders do advise The National Archives but there might be merit in a working group or other actions to better surface the issues around selection choices for personal data.

 Having made a number of points relating to the long-term value of a range of type of records, the letter raises concerns surrounding data protection misunderstandings. This is an important component of the piece. The full complexity and risks around this could have been further developed. The letter makes the case that there does need to be better understanding of the legal rights to retain information for archival/historical research purposes. Under the new General Data Protection Regulation (GDPR)/Data Protection Act 2018 regime there is additional complexity in the legislation as there is a recognition of the need to retain personal data for historical and research purposes and in addition recognition of the value of archiving in the public interest. This is an important new layer in the legislation which strengthens the case for the long-term retention of personal data, the value of historical research and the role of archives. It is right that the message that personal data can be kept does need to be communicated and disseminated as widely as possible. The current operational, archival, and historical considerations for retaining personal data do influence record storage and management plans through time. The authors highlight a very real concern that organisations may not properly balance and weigh the full range of considerations as to whether or not to retain personal data. Whilst a significant focus of this piece relates to UK Government records, in fact potentially the biggest risks that records will be lost is likely to be in contexts outside of the public authority domain where there is no requirement to have archival processes in place, e.g. in terms of business and charities where accountability and data use through the longer term will often not be a fundamental consideration. As such, I agree with the authors that the risk of data destruction is ‘re-emerging’ and has been heightened by the new legislation. Under data protection law, if an organization is the target of a security attack, it will subsequently be scrutinized not only as to whether or not it took appropriate security measures but in addition, whether any of the personal data compromised could have been destroyed at an earlier point in time thus minimizing the scale and impact of the attack. This position is evidenced in the recent case of the credit agency Equifax, which had a cyber security breach that compromised the details of millions of global citizens. This breach has been public knowledge for some time but the ICO has issued a £500,000 fine since the publication of this letter (ICO, 2018 ^3^). The ICO make the point that Equifax had retained personal data beyond its operational requirement for this data and as such it could have been deleted/purged. The security breach occurred before the introduction of new data protection laws. Had the same breach occurred under new the General Data Protection Regulation (GDPR)/Data Protection Act 2018 regime the financial penalty could have been far higher. Organisations will be taking note of this and other such decisions and reviewing pending retention/disposition schedules in the light of this ruling. From a data protection risk management perspective this will encourage organisations to destroy personal data far earlier.  In addition, individuals now have some rights of control over their data as there is now a ‘right to be forgotten’ in certain specific circumstances although not if the record/data is ‘archived’. As such accidental survival is less likely as rightly organisations must manage their personal data. Equifax are not likely to have considered long term historical/archival factors in terms of data retention, but credit data will have some longer term value. The question is how best we decide what is of value and encourage organisations to serve the interests of individuals/customers taking into account societal needs through time. Organisations do need to be made aware of the need to review the retention of data with longer term considerations in mind. Archivists and research communities must articulate stronger calls for archival retention targeting and explaining the value of particular personal data information far sooner, if this information is to be retained. The case for retaining medical records has been well evidenced and seems to be widely understood but the same case has not been made for many other personal records/data sets with valuable cultural, social and economic information.

 In terms of providing guidance in the area of data protection law, archivists are taking action although more can be done. The National Archives does have an FAQs page on GDPR http://www.nationalarchives.gov.uk/archives-sector/advice-and-guidance/managing-your-collection/archives-data-protection-law-uk/gdpr-faqs/ . Stronger links could be made between the ICO and TNA guidance. Significantly The National Archives has produced a *Guide* *to archiving personal data* which clearly states:

 “the law recognises there is a public interest in permitting the permanent preservation of personal data for the long-term benefit of society” (TNA, 2018, p.6 ^4^)

 In addition, the *Guide* has been supported by the ICO and has a foreword by the Information Commissioner, Elizabeth Denham, supporting the place of archives:

 “Archives are special places. They are our collective memory. They help us to understand the past, make sense of the present, and guide us for the future. And in an age of fake news, misinformation and opaque institutions, archives are more important than ever in helping to uphold democracy and hold power to account.”  (TNA, 2018, p.4 ^4^)

 The Archives and Records Association ( https://www.archives.org.uk/) is in the process of producing more detailed guidance on the application of data protection law.

 In summary this letter does present a timely perspective on the issues arising from the implementation of data protection law.  It is to be hoped that authors will continue to advocate to raise awareness of the concerns around potential data loss and the value of personal data  more generally. If there were any more specific recommendations that could be made within the letter, then this would be beneficial. However, whilst data protection law has brought risks one thing GDPR and global digital developments have achieved is to generate a better understanding of the value and potential uses of personal data which has perhaps not been fully recognised in archival collection strategies. I hope the authors will continue to evolve this discussion and applaud the timely publication of this piece.

I have read this submission. I believe that I have an appropriate level of expertise to confirm that it is of an acceptable scientific standard.

## References

[1] KershawR: Empire Windrush – 70 years on. *The National Archives*.2018; Reference source

[2] Records collection policy. *The National Archives London: OPSI*.2016; Reference source

[3] Equifax Ltd: monetary penalty notice, 19th September 2018. *ICO*.2018; Reference source

[4] Guide to archiving personal data. *The National Archives London: OPSI*.2018; Reference source

# Peer review: Referee response for version 1

**DOI:** 10.21956/wellcomeopenres.16124.r33873

**Published:** 2018-09-24

**Authors:** Natalie Banner

**Affiliations:** 1 Understanding Patient Data Lead, Wellcome, London, UK

This Open Letter raises a timely issue about the risk of historical records, that have high potential value for research, being destroyed by over-cautious interpretation of new data protection legislation. The destruction of the ‘Windrush’ generation disembarkation cards is a clear case in point even though this occurred prior to the EU General Data Protection Regulation (GDPR) coming into force: these records could have provided a rich resource for retrospective research.

 The introduction of the GDPR has thrown issues about data protection and retention into sharp relief among the research community, not least because of the high monetary penalties for failure to comply. It is also a complex piece of legislation, made more difficult to navigate in the UK context because of the UK’s domestic legislation having to perform a balancing act between pre-Brexit compliance and post-Brexit independence.

 The authors are wise to focus in on one particular aspect of this complexity and to explore its implications for the specific but vitally important area of longitudinal research. The examples they draw on demonstrate clearly the value of using historical records for retrospective research, and set out why this kind of research may be at risk.

 The letter needs updating in light of the UK Data Protection Act (2018) now being in force. As the GDPR Article 89(1) safeguards are derogated to Member States to specify, it would be helpful to include reference to the specific text used in the DPA (s.19)  to describe these safeguards in the UK context. It is imperative that the conditions necessary to fulfil these safeguards are more widely understood among the research community as they provide:

1. the conditions under which data can be retained for archiving purposes, as the authors correctly note, but also
2. the only lawful route through which research with special categories of personal data (including data about health, racial or ethnic origin, genetics etc) can be conducted on a “public interests” lawful basis (via Article 9(j) of the GDPR)

In my view, disseminating understanding about the demands of the Article 89(1) safeguards needs to go beyond the audiences the authors identify: it is not only a matter for operational record managers and archivists, but for all researchers who use special category data in their research. I therefore consider that the authors have correctly diagnosed a significant concern but also underappreciated its potential impact beyond questions of long-term retention.

 The recommendations are a helpful and concrete specification of actions needed from different stakeholders to rectify the lack of clarity about what the GDPR actually requires for long-term record retention. However, this is an area replete with guidance that is not always consistent and that can add to confusion, especially when compounded with differing institutional and funder policies. Rather than the ICO (the UK’s data protection regulator) issuing further guidance, further work to understand the specific areas of misunderstanding at a community and practice level, across research institutions, records offices, archives etc, may be a more fruitful starting point.

 Overall, this Open Letter starkly illustrates a tangible risk emerging from misinterpretation of GDPR conditions for data retention, and one that should be drawn to wider attention among the longitudinal research community. The call for further public engagement and discussion about the value of historical records is encouraging, given the broader context of research participants and the public becoming increasingly aware of data about them being collected by various organisations for numerous purposes, and about their rights as data subjects. Research can and should be held to high standards of fairness, transparency and accountability and this letter is a step towards enabling a clear public rationale for why personal data should be retained for archiving purposes in the public interest.

I have read this submission. I believe that I have an appropriate level of expertise to confirm that it is of an acceptable scientific standard.
